# Neural Correlates of Empathy in Boys With Early Onset Conduct Disorder

**DOI:** 10.3389/fpsyt.2020.00178

**Published:** 2020-03-18

**Authors:** Georg G. von Polier, Ellen Greimel, Kerstin Konrad, Nicola Großheinrich, Gregor Kohls, Timo D. Vloet, Beate Herpertz-Dahlmann, Martin Schulte-Rüther

**Affiliations:** ^1^Brain & Behaviour (INM-7), Research Centre Jülich, Institute of Neuroscience and Medicine, Jülich, Germany; ^2^Department of Child and Adolescent Psychiatry, Psychosomatics and Psychotherapy, Medical Faculty, RWTH Aachen University, Aachen, Germany; ^3^Department of Child and Adolescent Psychiatry, Psychosomatics and Psychotherapy, University Hospital, LMU Munich, Munich, Germany; ^4^Child Neuropsychology Section, Department of Child and Adolescent Psychiatry, Medical Faculty, RWTH Aachen University, Aachen, Germany; ^5^JARA-Brain Institute II, Molecular Neuroscience and Neuroimaging, Research Center Jülich, Jülich, Germany; ^6^Department of Social Sciences, Institute of Health Research and Social Psychiatry, Catholic University of Applied Sciences of North Rhine-Westphalia, Cologne, Germany; ^7^Department of Child and Adolescent Psychiatry, Psychosomatics and Psychotherapy, Centre of Mental Health, University Hospital of Würzburg, Würzburg, Germany; ^8^Translational Neuroscience in Psychiatry and Neurology, Department of Child and Adolescent Psychiatry, Medical Faculty, RWTH Aachen University, Aachen, Germany

**Keywords:** affective empathy, cognitive empathy, amygdala, medial prefrontal cortex, callous-unemotional traits, psychopathy

## Abstract

**Background:** A deficit in empathy has repeatedly been described in individuals with conduct disorder (CD), and in particular in those with callous unemotional traits. Until now, little is known about the neural basis of empathy in children and adolescents with early onset conduct disorder. The aim of this study was to examine neural responses during empathizing in children and adolescents with CD with a task that allowed to differentiate between the judgment of the emotional states of other people and the own emotional response to other people's emotional state. Moreover, we investigated associations of callous-unemotional traits and neural activations during empathizing.

**Methods:** Using functional magnetic resonance imaging (fMRI) we investigated 14 boys with early onset CD and 15 typically developing (TDC) age matched controls between 8 and 16 years of age. Happy and sad faces were presented, and participants were asked to either infer the emotional state from the face (other-task) or to judge their own emotional response (self-task). A perceptual decision on faces was used as a control task. Individual empathic abilities and callous unemotional traits were assessed.

**Results:** During the other task, TDC boys showed significantly larger right amygdala responses than CD boys. Higher empathic abilities (as assessed with the Bryant Index of Empathy) were associated with higher responses in the right amygdala within the CD boys and across the entire sample. Moreover, across the entire sample, callous-unemotional traits were negatively related to the BOLD-response in the right amygdala. CD boys showed larger responses in the dorsal and ventral medial prefrontal cortex across tasks and increased activation in dorsal medial prefrontal cortex specifically during the self-conditions, which were also related to empathic abilities within the CD boys.

**Conclusions:** The data emphasize the important role of the amygdala in empathy related emotional processing. Diminished amygdala responses and their association with low empathy suggest a pivotal influence of impaired amygdala processing in early-onset CD, in particular for deficits in empathic behavior and related callous-unemotional-traits. Elevated response in the medial prefrontal cortex in boys with CD point toward increased involvement of brain areas related to self-referential processing and cognitive empathy during empathizing.

## Introduction

Conduct disorder (CD) is a serious neurodevelopmental disorder characterized by a repetitive and persistent pattern of disruptive behavior that violates the basic rights of others and major age-appropriate social norms or rules (1). It is one of the most frequent psychiatric disorders in childhood and adolescence resulting in referral to mental health services (2). CD is accompanied by mental and physical health problems and negative psychosocial outcomes with an increased risk for lifelong antisocial behavior, resulting in considerable healthcare, and societal costs (3). However, children with CD are a strikingly heterogeneous group with respect to clinical presentation and outcome (4). Children and adolescents with an early onset CD (before age 10) show a particularly poor prognosis with frequent development of subsequent criminality, substance abuse and antisocial personality disorder (5, 6). A second criterion to differentiate CD—callous unemotional (CU) traits—was added to the Diagnostic and Statistical Manual of Mental Disorders [DSM-5, (1)]: the lack of empathy and guilt, callousness, and uncaring attitudes. CU-traits are associated with an early-starting and chronic trajectory of disruptive behavior with especially unfavorable developmental consequences (7). While CD without CU traits appears to be more strongly related to environmental risk factors, CD with high CU traits seems to have a greater biological foundation (8, 9) and high CU traits are associated with an early onset of the disorder (10).

Accordingly, theoretical models of CD etiology have proposed that dysfunction in different neurocognitive systems may be associated with different types of CD-related symptom sets (11), including CU traits [for a recent review, see (12)]. Accumulating evidence indicates that children with CD but low levels of CU traits typically show a heightened affective reactivity to perceived negative emotional stimuli, such as angry or ambiguous neutral facial expressions, which may be regarded as social threat or provocation, resulting in reactive aggressive acts (13). In stark contrast, a reduced affective responsiveness to others' distress signals, such as fearful or sad facial expressions, appears to be a characteristic dysfunction of CD individuals with high levels of CU traits. Such a profile is thought to contribute to a repertoire of rather proactive aggressive behavior that harm other people (e.g., violence) (14, 15). Thus, the latter form of CD is assumed to constitute a group of individuals with decreased emotional empathy, which involves a marked difficulty in decoding and representing the emotional states of other people.

Converging lines of research using functional magnetic resonance imaging (fMRI) have consistently demonstrated that higher CU traits among individuals with CD problems are particularly associated with reduced amygdala activation in response to distress cues, including fearful and sad facial expressions as well as witnessing other persons in pain (11, 16–20). While the vast majority of relevant fMRI studies has investigated rather basic affective responsiveness in youth with CD (i.e., recognizing simple emotion expressions), a minimal amount of work has more directly investigated higher-level empathic functioning (i.e., inferring the emotional state of others or one's own emotional response). Importantly, reduced empathy is a key criterion for identifying high CU traits and both traits are inversely related. Sebastian et al. (21) were the first to demonstrate amygdala hypoactivation (accompanied by reduced anterior insula cortex activation) in children with CD problems in the context of an empathy paradigm that required participants to infer how a story character would react to their companion's affective state through a series of pictorial story vignettes. Importantly, the authors found suppressor effects such that amygdala reactivity decreased as a function of CU traits but increased as a function of conduct problems. However, this study investigated a community sample with conduct problems but not a clinical sample with a diagnosis of CD. Other fMRI-studies of explicit empathizing in participants with psychopathic traits likewise point toward diminished neural responses in comparison to healthy participants (22–24). Moreover, these studies suggest that patients with psychopathic traits may have the ability to empathize with other people but are less inclined to use this ability during social interaction (23). Youth with CD and particularly those with high CU-traits appear to have deficits concerning affective responses relevant for empathy (13), but do not show impairments in cognitive aspects of empathy such as mentalizing or theory of mind (21, 25). Thus, a possible pathway to understand the ability to empathize in individuals with high CU-traits could be an increased use of strategies related to cognitive empathy.

Cognitive empathy involves the representation of thoughts and intentions of other individuals and the process of mentalizing (26). Mentalizing and theory of mind has typically been associated with activation of the medial prefrontal cortex (MPFC), also in the context of empathizing (26). While studies on explicit empathy tasks focusing on cognitive aspects (21) have not found dysfunctions in the MPFC in CD, other studies using resting state fMRI suggests functional alterations in this brain region in individuals affected by the disorder (27, 28), which the authors interpreted as reflecting deficient introspective processing, emotion processing and reduced empathy. Therefore, the authors recommended investigating correlates of cognitive empathy and deficient introspective processing in more detail.

In summary, while previous research has established a blunted amygdala reactivity, this has rarely been investigated using higher-level empathy tasks in youth with CD. Second, while CD individuals are able to empathize in order to achieve goals or when explicitly instructed (22), they still show deficits in processing sad and fearful faces, show little compassion, a shallow affect and lack of guilt, in particular with high CU traits (29). A detailed knowledge about neural mechanisms related to empathizing in CD, including cognitive and affective aspects, is lacking. This is of particular interest in early-onset CD, as this subtype is thought to be characterized by marked neurodevelopmental disturbances and a higher risk to develop CU-traits (10). Given the poor prognosis and very limited treatment options of early-onset CD particularly with high CU-traits (30), it is of great scientific and clinical interest to understand neural mechanisms of empathy in this cohort but has not been studied to date. Due to the relevance of reduced empathy for the concept of psychopathy and CU traits (31), and strong evidence for negative associations between individual empathy and CU-traits (32), we aimed for a combined investigation of neural substrates of empathy, individual empathy measures and CU-traits. We employed an established explicit empathy tasks, which involved a face-to-face situation and draws on both cognitive and affective aspects of empathy. This task requires participants to empathize with emotional faces by (a) inferring the emotional state of others as expressed by their facial expression (other-task) and (b) judging the own emotional state in response to the depicted facial expression (self-task). Thus, this task draws on evaluating someone's emotion, taking the self-perspective, and focusing on own evoked emotions. We investigated a clinical sample of early-onset conduct disorder (i.e., presence of at least one characteristic CD behavior prior to age 10, according to DSM-5), known to show severe antisocial behavior and high levels of callous-unemotional traits (33). We were particularly interested in neural activation of the amygdala, given its pivotal role in affective empathy processing and the history of studies indicating dysfunction in CD. In line with earlier research, we hypothesize a blunted amygdala response also during explicit empathize tasks. We also focused on neural activation within the MPFC in line with its pivotal role for cognitive aspects of empathy. Moreover, we expected a positive relationship between individual empathic abilities and brain activation in brain areas related to empathizing, and, accordingly, an inverse relationship with CU traits (32).

## Materials and Methods

### Participants and Diagnostic Assessment

The final sample for the fMRI analysis comprised 14 boys with early onset CD between 8 and 16 years of age, and 15 TDC boys (see below for excluded participants after fMRI data analysis). Only subjects with an IQ ≥ 80 based on the Wechsler Intelligence Scale for Children-IV (WISC-IV, German version by (34) were included. Both groups were age-matched, however TDC showed slightly higher IQ (see Table 1). Participants of the CD group showed significantly higher CD traits and lower empathy abilities.

**Table 1:** Sample description.

	**TDC (*n* = 15)**	**CD (*n* = 14)**
Age	12.7 (2.5)	12.2 (1.9)
Age range	8.5–16.8	8.1–14.6
Full-scale IQ (WISC-IV)	112.8 (10.5)	99.8 (14.2)^*^
IQ range	80–133	80–119
BIE (max. 88)	19.4 (16.2)	−0.3 (14.4)^**^
Callous-unemotional (APSD, T-score)	46.4 (7.9)	64.1 (7.6)^**^

Means and standard deviations given if not noted otherwise. CD, Conduct disorder; TDC, typically developing controls; BIE, Bryant Index of Empathy; APSD, Antisocial process screening device;

* p < 0.05;

** *p < 0.01*.

Participants with CD were recruited from the Department of Child and Adolescent Psychiatry of the RWTH Aachen University. Participants in the healthy control group were recruited by announcements in local schools. All participants were informed in detail about the experimental procedures and the aims of the study and provided written informed assent. Written informed consent was obtained by parents/legal custodian, after the parent(s)/legal custodian(s) had been informed about all aspects of the study. The study was approved by the local ethics committee in accordance with the Declaration of Helsinki and in compliance with national legislation.

Participants were included, if they did not have evidence for a neurological disorder, or a history or current diagnosis of psychosis, trauma, bipolar disorder, substance abuse, or pervasive developmental disorder based on a standardized semi-structured interview (K-SADS-PL) (35). Further exclusion criteria were any chronic physical illness and the use of any medication at the time of fMRI measurements. Medication with methylphenidate (*n* = 8) was stopped 48 h prior to the fMRI assessment.

Using the standardized semi-structured interview (K-SADS-PL) (35) with participants and caregivers, all participants were assessed for current and past CD, oppositional defiant disorder, ADHD, major depressive disorder, anxiety disorders, obsessive-compulsive disorder, tic disorder, elimination disorder, and posttraumatic stress disorder. The K-SADS-PL was also applied in TDC participants to rule out any current and past psychiatric condition.

Participants were included in the patient group if the diagnostic criteria of CD, early-onset subtype according to DSM-5 were fulfilled, i.e., if at least one symptom had started before the age of 10. Psychiatric comorbidity in the CD group was as follows: 10 participants (71%) were diagnosed with comorbid ADHD, two participants with comorbid enuresis, and one participant with comorbid chronic motor tic disorder. Cognitive testing (IQ) was performed in all participants using the WISC-IV, German version by (34).

### Instruments Assessing Behavioral Characteristics

Psychopathic traits in the CD and TDC groups were measured by the Antisocial Personality Screening Device [APSD (36)], a 20-item rating scale that assesses callous-unemotional (CU) traits, narcissism and impulsivity. All parents completed a German version of the APSD, and the CU traits subscale was evaluated. Standardized values (T-Scores) were used, provided in the manual (36).

Empathic abilities: All children completed the German version of the Bryant Index of Empathy (BIE); (37, 38), an adaptation of the Emotional Empathic Tendency Scale (39) assessing predominantly affective aspects of empathy. Higher scores on the BIE reflect greater empathic ability. Items are rated on a 9-point Likert scale (−4 to +4); the maximum total sum score is 88.

ADHD symptoms: Parents completed the German Parental Report on ADHD symptoms, which is part of the Diagnostic System of Mental Disorders in Children and Adolescents (40) reflecting DSM-IV criteria. Sum scores are provided.

### Stimuli

**Figure 1:**
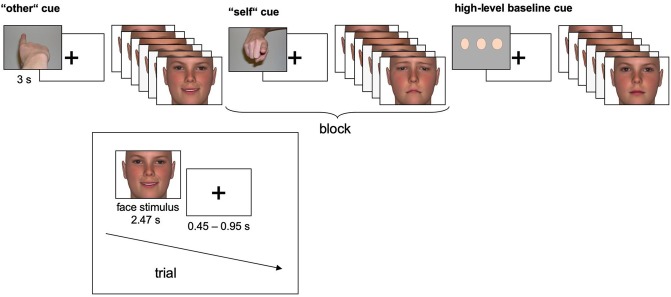
Experimental time course. In the self- and other-task, happy and sad faces were presented, and participants assessed which emotional state (sad, neutral, or happy) best described the emotional state of the other person (other-task) or their own emotional response to the face (self-task). In the high-level baseline, subjects judged the width of neutral faces (thin, average, or wide). Each of *n* = 72 individual faces was presented once displaying a happy expression, once displaying a sad expression and once displaying a neutral expression.

The facial stimuli were taken from an earlier study of our group, published in (41–43). In brief, happy, sad and neutral faces (*n* = 72, each) were used as stimuli. Happy and sad expressions were chosen since they have been shown to evoke congruent empathic reactions (e.g., feelings of sadness when viewing a sad face) (44). Since empathic feelings are more easily evoked when the counterpart is similar to oneself (e.g., in age and gender) (45), a set of face stimuli was constructed and morphed to the age of the participant groups. Furthermore, the stimuli were morphed to neutral and emotional faces according to established conventions (Facial Action Coding System) (46) using FaceGen 3.1 (Singular Inversions, Vancouver, Canada). Weak emotional expressions were used along with more obvious ones to provide sufficient variability with regard to emotional intensity (see Figure 1).

### fMRI Paradigm

Three tasks alternated blockwise in a pseudo-randomized, counterbalanced order (see Figure 1 for details). In the other-task, participants were instructed to empathize with a person whose face appeared on the screen and to infer the emotional state from that face. In the self-task, participants were again instructed to empathize with the depicted person. However, they were asked to *judge their own emotional response* to the face. In both tasks, happy and sad faces were mixed within blocks to prevent habituation effects. Half of the blocks contained high-intensity emotional stimuli, and the other half contained low intensity emotional stimuli. Response options were “sad,” “neutral” or “happy”. In the high-level baseline task, perceptual decisions on the width of neutral faces as “thin,” “average,” or “wide” were included. Participants responded with their right index, middle, and ring fingers using a three-button response device. Each block (19.2 s) was preceded by an instruction cue (3 s) and comprised six face trials (each 2.47 s), separated by a fixation cross (0.45–0.95 s). 10 blocks of each task were presented, resulting in 30 blocks.

To familiarize participants with task requirements, participants first practiced the task outside the scanner. To reduce potential bias in the self-report of own emotions (self-task), we explicitly instructed the participants that there was no “wrong” answer and they should respond according to their actual feelings. Faces shown during the practice session were not included into the fMRI stimulus set. After the fMRI experiment, participants were asked standardized open questions on how they resolved the tasks. All participants were able to recall and describe how they resolved the tasks. Participants who did not follow the task instructions correctly (e.g., stated that they judged the emotional state of the person whose face appeared on the screen during the self-task instead of judging their own emotional response to the face) were excluded from the sample; this applied to one TDC participant.

Seventeen boys with early onset CD and 18 typically developing controls (TDC) boys were investigated. After exclusion of six participants (3 CD, 3 TDC) due to excessive movement (see below for details), 14 CD and 15 TDC boys remained for the final analysis. Response collection and stimulus presentation were performed employing the software Presentation 9.9 (Neurobehavioral Systems, Albany, CA, USA). Visual stimuli were presented using a head mounted display.

### MRI Acquisition

Scanning was performed on a 3.0-Tesla Trio-Tim system (Siemens, Erlangen, Germany) using a standard CP head coil. During the experimental task (11.8 min), whole brain echoplanar T2-weighted images (EPIs) were acquired (TE = 28 ms, TR = 2,230 ms, FlipAngle = 77°, FOV = 192 mm, matrix size = 64 × 64, 38 slices, slice thickness = 3 mm, voxel size = 3 × 3 × 3.45 mm^3^). High-resolution T1-weighed anatomical images were collected using a rapid acquisition gradient-echo (MP-RAGE) pulse sequence (TE = 2.520 ms, TR = 1.900 ms, FlipAngle 9°, FOV = 256 mm, matrix size = 256 × 256, 176 slices, slice thickness = 1 mm, voxel size = 0.98 × 0.98 × 1 mm^3^).

### Data Analysis

Imaging data was analyzed using SPM12 (Wellcome Trust Centre for Neuroimaging, London, UK, http://www.fil.ion.ucl.ac.uk/spm/) implemented in MATLAB 8.4 (The Mathworks, Inc., Natick, MA, USA). The first four functional images of each participant were discarded. The remaining 312 volumes were realigned, spatially normalized to standard stereotactic Montreal Neurological Institute (MNI) coordinates and spatially smoothed with an 8-mm full-width half-maximum Gaussian kernel. Participants with excessive movement (i.e., >3 mm translation, > 4° rotation) were excluded from further analysis resulting in the final sample size described in the participants section. Additionally, we checked that the overall amount of movement was not significantly different between groups by comparing respective max translation and rotation parameters across groups (multiple t-tests, *all t* < 0.84, all *p* > 0.41). Model parameters were estimated for each voxel according to the General Linear Model. To account for individual residual movement-related variance, realignment parameters were included into the model as regressors. For group analyses, a second-level random-effects model was implemented. Individual contrast images coding for each experimental condition were analyzed using a flexible ANOVA model with group as a between subject factor, and condition as within subject factors. Anatomical images were coregistered to the mean EPI image and normalized into MNI space. Boxcar functions (19.2 s, corresponding to the experimental conditions) were convolved with a model of the hemodynamic response and its first-order temporal derivative. Violations of sphericity assumptions were accounted for by applying the non-sphericity corrections in SPM12 (estimation of covariance components). As initial analyses did not reveal differential significant group effects for the comparison of high- vs. low intensity conditions (or vice versa), we focused on tasks and task x group interactions for the following analyses. For between-group comparisons (CD vs. TDC), we compared other- and self-blocks (containing both high and low intensity stimuli), with the high-level baseline, respectively (other > high-level baseline; self > high-level baseline) and performed the direct comparison between self- and other-tasks (self > other, other > self).

Results are reported that met the statistical threshold of *p* < 0.05 family-wise error (FWE) at the voxel level. For a-priori specified regions, we performed additional region of interest (ROI) analyses, i.e., the amygdala, and the medial PFC using a threshold of *p* < 0.05 (FWE corrected across each particular region). ROIs were constructed using standard neuroanatomical toolboxes implemented in SPM12 [Anatomy Toolbox (47), WFU PickAtlas (48)]. In detail, the ROIs for the left and right MPFC were constructed with regions of the aal-atlas including the cortices frontal superior medial, frontal medial orbital, gyrus rectus and frontal superior orbital.

Moreover, to assess whether brain activity was related to measures of empathy and callous-unemotional traits, linear regression analyses were performed with empathy scores (BIE/CU-traits respectively) for those regions and contrasts which yielded group differences between tasks. For regression analyses across and within groups, anatomical ROIs were used (amygdala, MPFC, thresholded at *p* < 0.05, FWE corrected for respective ROI). Additionally, we used a 15 mm sphere around coordinates with a significant group difference to further explore correlations within the CD group.

## Results

### Behavioral Results

Reaction times (RT) were analyzed by a 2 × 2 × 2 mixed-model ANOVA with the factors task, emotion, and group. This analysis did not yield significant main effect of task, emotion or group (all *ps* > 0.25). Moreover, interactions involving the factor group were all non-significant (all *ps* > 0.28).

Correct identification of displayed emotions during empathizing was assessed using correct responses during the other-task and analyzed by a 2 (emotion) × 2 (group) mixed effects ANOVA. We found a significant main effect of emotion *F*_(1, 27)_ =12.06, *p* < 0.01 due to a higher accuracy for sad (*M* = 82.5%; *SD* = 19.2) compared to happy faces (*M* = 71.0%; *SD* = 16.5; *p* = 0.002). The interaction of emotion x group was not significant [*F*_(1, 27)_ = 1.14] and there was no main effect of group [*F*_(1, 27)_ = 0.211].

Congruence of evoked emotions was assessed using congruent responses during the self-task (i.e., happy responses to happy faces and sad responses to sad faces) and analyzed by a 2 (emotion) × 2 (group) mixed effects ANOVA. We found a significant interaction of emotion × group [*F*_(1, 27)_ = 6.88, *p* < 0.05], but no main effect of emotion or group (all *ps* > 0.63). Post-hoc comparisons indicated a significantly higher congruency for sad (*M* = 64.3%; *SD* = 32.4) compared to happy faces (*M* = 51.4%; *SD* = 27.4; *p* < 0.05) in TDC but not in CD. On a descriptive level, this pattern was reversed in CD (sad: *M* = 44.2%; *SD* = 38.2; happy: *M* = 60.8%; *SD* = 29.2). Additionally, we performed the same analyses including intensity as a factor in the ANOVA-models (2 × 2 × 2 mixed-model ANOVAS for the factors intensity, emotion and group). In these analyses, no additional significant effects of group or interactions with group were observed, but all effects mentioned in the main manuscript remained stable (see Supplementary Material for further details). CD-Patients had significantly higher CU-traits (*t* = −5.9, *p* < 0.001) and significantly lower BIE-scores than TDC (*t* = 3.5, *p* = 0.001). Both measures were inversely related across groups (*r* = −0.5, p = 0.007).

### fMRI Results

Comparisons related to the main effect of task (i.e., self/other vs. control condition) yielded widespread activation in brain areas previously associated with empathic processing (41, 43, 49, 50) across both groups, including the amygdala, MPFC, middle temporal gyrus, temporal gyrus, inferior frontal gyrus, cingulum, and precuneus. For details see Supplement 1.

Whole brain comparisons or any contrast associated with group comparisons or interactions with the factor group did not yield significant activation peaks (FWE corrected threshold) for any task. Inference with respect to the factor group is drawn based on ROI analysis as outlined in the introduction and methods section.

#### Other vs. High-Level Baseline

**Figure 2:**
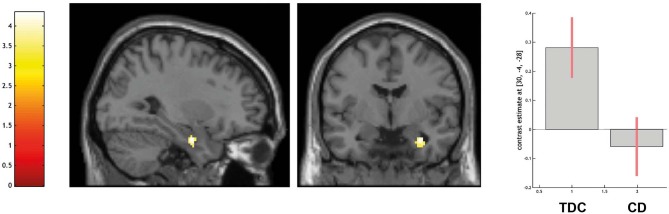
Differential activation of typically developing controls (TDC) and youth with conduct disorder (CD) for the contrast other > high level baseline, SPM(T) overlaid on single subject T1 template, threshold for illustrative purposes *p*_uncorr._ < 0.001. Increased activation in the right amygdala (peak coordinate 30, −4, −28) in TDC > CD (family-wise error corrected at *p* < 0.05 for ROI). Contrast estimates and 90% confidence intervals of contrast other > control in peak coordinate displayed. The color bar chart represents *T*-values.

ROI-based analyses of the amygdala confirmed that TDC yielded greater activation than CD in the right amygdala (peak voxel coordinates = 30, −4, −28; *t* = 4.02; *P* < 0.05 FWE-SVC; see Figure 2).

Across the entire sample, greater activation in the amygdala was associated with a higher degree of empathy (as indicated by higher BIE-scores, peak voxel coordinates = 30, −4, −26; *t* = 4.33; *P* < 0.01 FWE-SVC) and with a lower degree of CU traits (as indicated by CU scores, peak voxel coordinates = 26, −2, −28; *t* = 3.65; *P* < 0.05 FWE-SVC).

Within the CD group, higher BIE-scores were associated with greater BOLD-response in the amygdala (peak voxel coordinates = 30, 0, −24; *t* = 6.85; *P* < 0.01 FWE-SVC), however no significant association emerged with respect to CU traits. No group differences occurred in the ROI-based analysis of the MPFC.

#### Self vs. High-Level Baseline

**Figure 3:**
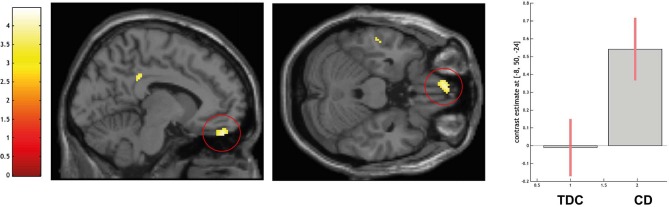
Differential activation of typically developing controls (TDC) and youth with conduct disorder (CD) for the contrast self > high level baseline, SPM(T) overlaid on single subject T1 template, threshold for illustrative purposes *p*_uncorr._ < 0.001. Increased activation in the left ventromedial prefrontal cortex (peak coordinate −8, 50, −24) in CD > TDC (family-wise error corrected at *p* < 0.05 for ROI). Contrast estimates and 90% confidence intervals of contrast self > control in peak coordinate displayed. The color bar chart represents *T*-values.

ROI-based analyses of the left MPFC indicated a higher activation in CD over TDC in the left ventromedial prefrontal cortex (peak voxel coordinates = −8, 50, −24; *t* = 3.95; *P* < 0.05 FWE-SVC; see Figure 3). No difference in amygdala activation was detected.

#### Self vs. Other Task

**Figure 4:**
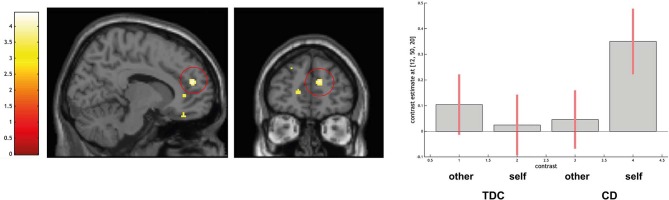
Differential activation of typically developing controls (TDC) and youth with conduct disorder (CD) for the contrast self > other, SPM(T) overlaid on single subject T1 template. Threshold for illustrative purposes *p*_uncorr._ < 0.001. Increased activation in the right dorsomedial prefrontal cortex (peak coordinate 12, 50, 20) in CD > TDC (family-wise error corrected at *p* < 0.05 for ROI). Contrast estimates and 90% confidence intervals of contrasts other > control and self > control in peak coordinate displayed. The color bar chart represents *T*-values.

ROI-based analyses for the comparison CD > TDC indicated no differences in the amygdala and larger activation in the right dorsal PFC (peak voxel coordinates = 12, 50, 20; *t* = 4.19; *P* < 0.05 FWE-SVC; see Figure 4).

Using ROI-based analysis with a sphere of 15 mm around the coordinates 12, 50, 20 a positive linear association with BIE-scores was detected in the CD group (peak voxel coordinates = 12, 52, 34; *t* = 5.37; *P* < 0.05 FWE-SVC). No difference in amygdala activation was detected.

Across all above-mentioned coordinates and contrasts no associations with IQ or ADHD-symptoms scores were detected.

## Discussion

The aim of this study was to examine neural correlates in an explicit empathizing task in early onset CD as compared to TDC. Key findings of this study include the hypoactivation of the amygdala during the other-task in CD participants. Amygdala activation was positively associated with measures of empathy in the whole sample and in the CD group alone. Moreover, during the self-conditions, CD showed larger activation specifically in the ventral and dorsal MPFC pointing toward increased activation in areas relevant for cognitive empathy and self-referential processing (51).

With respect to behavioral performance, there were no significant differences in RTs between CD participants and control participants for any experimental condition. It is thus unlikely that differences in neural activations are related to domain-general performance deficits (such as differences in perceptual processing speed).

Moreover, consistent with other reports using a similar paradigm (24), our behavioral findings (i.e., task-performance) do not indicate deficits in CD for the identification of other people's emotional state (other task) during empathizing. While other studies have reported deficits for emotion recognition in CD (18, 52, 53), this does not appear to be the case in the context of empathizing with clearly identifiable facial expressions. However, despite comparable emotion *identification*, we could demonstrate atypical emotional *resonance* (self-task) in CD participants, revealing that CD participants do not show the same increased resonance with sad emotions as evident for TDC. Such a specificity for the displayed emotion is consistent with other reports of sad-specific impairments in antisocial individuals (18) for emotion processing. Furthermore, this pattern of results resonates well with the clinical characteristics of early-onset CD and in particular high CU traits. As outlined in the DSM-5, individuals with limited prosocial emotions show lack of remorse e.g., “after hurting someone” (1), and are less concerned about the feelings of others, or disregard others feelings. This aberrant pattern may be connected with a less empathic reaction to others' emotions and in particular to sad faces. A limited resonance with sad expressions as an example for a distress cue has been associated with the theory of reduced cognitive violence inhibition (13, 54). This theory suggests that a victim's distress cues can inhibit the attacks of an aggressor. In line with a limited responsiveness to distress cues or reduced violence inhibition, children with early onset CD often show an early onset of severe aggression (55).

The amygdala plays an important role for emotional processing, including empathy [see (56, 57) for recent meta-analyses]. Robust involvement of the amygdala across participants confirms this also in the context of the empathy task that we used (41, 58). Furthermore, we could demonstrate a correlation of individual empathic abilities and brain activation with the amygdala, underlining the importance of the amygdala integrity for empathic responses. Blunted amygdala activity has repeatedly been reported in the context of psychopathic traits and antisocial behavior in response to fearful faces (16, 21, 59, 60). Our finding of a hypoactivation of the right amygdala in early-onset CD is consistent with previous reports on reduced amygdala reactivity specifically for sad vs. neutral faces (20). Of note, the study of Passamonti et al. (20) revealed that this amygdala hypoactivation was more pronounced in early onset than adolescent onset CD. Our study is the first to reveal hypoactivation in the context of empathizing in early onset CD, and associations with CU traits and empathic abilities. Moreover, several studies point toward amygdala hypoactivation as an important neurobiological marker of reduced pain-related empathy and responses to distress in psychopathy in adolescents and adults (59, 61). While earlier studies on emotional processing in CD have demonstrated links between amygdala activation and callousness (16, 17), our study is the first to report an association between amygdala activity and empathy even within a CD group using a measure of individual empathic abilities (i.e., the BIE). This finding highlights that despite overall impairment, there is considerable variability of empathic abilities in CD participants which is associated with amygdala responsivity during empathizing. Although we found an inverse association between amygdala activity and CU-traits and, accordingly, an inverse relationship between empathy and CU traits across the whole sample, we could not demonstrate an association of amygdala activity and CU-traits within the CD group. This was likely due to the fact that the CD participants in our study had relatively high CU values with low variance. Further studies with larger sample sizes are needed to address the complex relationship between empathy, CU-traits and brain activation patterns.

With respect to (frequently) comorbid ADHD diagnoses in CD, our data did not point toward associations of amygdala activity and ADHD comorbidity in accordance with a recent meta-analysis (62). In summary, our finding of a blunted amygdala reaction in an empathy task in early onset CD adds to the body of literature which argues for a biological basis as an explanatory model for a high risk to show pervasive antisocial behavior (5).

During the self-task, as well as for the direct comparison of self vs. other task, we observed higher activation in the MPFC for the CD group (as compared to TDC). Brain activation in the MPFC has consistently been reported for diverse social tasks, including mentalizing and cognitive as well as affective empathy (49, 50, 56, 57, 63). A bulk of evidence converges on the finding of a gradual ventral-dorsal distinction within the MPFC with more dorsal aspects implicated for goal-directed behavior, cognitive control, and social-cognitive judgments, whereas ventral aspects are more associated with self-relevance in emotional contexts (such as autonomic responses and monitoring the evaluation of future outcomes) (64). Similarly, dorsal and ventral MPFC can be conceived as representing a functional gradient from more involvement for other-related judgments (dosal) to self-related judgments (ventral) (51). Accordingly, vMPFC has also been implicated in self-referential emotional cognition [e.g., (65)]. The anterior rostral part of the MPFC (arMPFC, in between the most dorsal and ventral parts) has been conceptualized as the central hub for more abstract metacognitive representations which support self-reference and mentalizing (64). This area is consistently activated during perspective taking, empathy, and theory of mind tasks (49, 50, 57, 66, 67). Our data reveal a greater involvement of the arMPFC (BA 32/10) for CD participants during explicit empathizing, in particularly when attending to the self-perspective. Others have suggested that areas such as the arMPFC are more related to cognitive aspects of empathy (66). Interestingly, increased brain activation in the arMPFC was also correlated with *better* empathic abilities in CD participants, suggesting that this area may, at least in part also be interpreted as serving a compensatory role (in the light of decreased emotional resonance paralleled by reduced amygdala activation). It might be speculated that compensatory MPFC activation could also play a role to enhance activation of the amygdala and enhance emotional resonance per se. However, this would need to be verified in future studies with larger samples to reveal a potential direct relationship between arMPFC, amygdala activation, and emotional resonance. Furthermore, we observed increased activation for the CD group (relative to TDC) of the vMPFC for self-related processing during empathy. This finding suggests a stronger reliance on self-referential processing during empathizing for CD, in concert with a less congruent emotional response. Taken together with the finding of reduced amygdala responses, this pattern of results resonates well with the clinical phenotype of callous-unemotional traits in patients with CD (i.e., self-centered, emotionally cold, and low empathic behavior toward other people).

Both arMPFC (BA 32/10) and vMPFC (BA 11) are part of the so-called default mode network (DMN), which is characterized by increased connectivity during periods of rest (68) and has been suggested to support emotional and self-referential processing (69). Two studies have reported reduced functional connectivity for CD patients in the DMN, and particular in MPFC (27, 28), also in the subgroup of early onset CD (27) which the authors interpreted as potentially reflecting dysfunctional introspective processing and hypothesized a relation to social-cognitive deficits in CD. Our data, however, suggest hyperactivation during an explicit self-task, in concert with a positive association with empathic abilities. Interestingly, a study investigating anatomical integrity of the DMN observed *increased* myelination in the DMN in adolescents with CD in comparison to TDC (70), which was also associated with CU traits. Clearly, future studies are needed to address these inconsistencies which combine resting state connectivity, anatomical connectivity and functional activation patterns and investigate the relation with CU traits and empathic abilities.

### Implications

Our data point toward increased brain activation in MPFC areas in CD known to be strongly involved in cognitive empathy, self-referential processing, and reward. Thus, in line with prior research, CD may have the ability to empathize (22), but rely on different neural mechanisms. The involved neural mechanisms may be less associated with emotional contagion, but possibly promote a more self-centered behavior displaying a shallow affect during social interaction.

A possible approach to improve empathy skills is the use of empathy training programs. A meta-analytic review on empathy training regimes indicates overall medium effect sizes of these programs (71). A study by Dadds (72) indicates that particularly boys with high CU traits may benefit from these programs in showing improved empathic skills and subsequently lower conduct problems. Future research could more closely evaluate if a targeted training of empathic skills could result in e.g., improved amygdala reactivity or differential neuronal processes underlying improved empathy in CD. Subsequently, a targeted fMRI-neurofeedback training in youth with low empathy could help improve the outcome of empathy-related trainings. Given that our study points to a certain variability of neural responses in the amygdala and the MPFC that were associated with empathy measures, an early and targeted beginning of empathy skills training could possibly help improving the outcome of an individualized treatment.

### Strengths and Limitations

Strengths of the study include the investigation of a well-characterized clinical sample with early-onset conduct disorder and the use of an established explicit empathy task that has been successfully used in prior research of our and other groups (24, 41–43, 49). In line with previous studies (42, 43, 49, 50) we interpret behavioral responses for the self-task as an index of emotional resonance. Although we cannot completely rule out the possibility that this self-report response could be biased (e.g., due to a tendency to respond in a socially acceptable way), our interpretation is in line with previous findings of reduced emotional contagion in CD (41–43, 49, 50). However future studies should consider using more objective measures of emotional resonance, e.g., skin conductance measures or video recordings of facial expression.

A limitation of the study is the limited sample size, which requires a replication of our results in larger studies. Furthermore, previous studies have also reported structural abnormalities in CD, also in similar regions where we found functional differences. In particular, early onset CD might be characterized by stronger neurodevelopmental disturbance, thus, structural and functional deficits may interact and both contribute to deficits in empathy processing and related brain areas. Future studies should systematically investigate structural and functional trajectories of brain areas related to empathy processing and CU traits across development in early onset CD.

## Conclusion

The data emphasize the important role of the amygdala in empathy related emotional processing in early onset CD during an explicit empathy paradigm. Diminished amygdala responses and their association with low empathy suggest a pivotal influence of impaired amygdala processing in early-onset CD, in particular for deficits in empathic behavior. Elevated response in the MPFC in boys with CD point toward increased demands on self-referential processing to solve empathy tasks, thus potentially pointing at a more cognitive biased processing strategy in this patient group. Future research may focus in more detail on neural correlates of cognitive empathy processing in CD and a possible improvement using empathy related trainings.

## Data Availability Statement

The datasets generated for this study are available on request to the corresponding author.

## Ethics Statement

The study was approved by the local ethics committee and carried out in accordance with the recommendations of good clinical practice. All participants were informed in detail about the experimental procedures and the aims of the study and provided written informed assent. Written informed consent was obtained by parents/legal custodian, after the parent(s)/legal custodian(s) had been informed about all aspects of the study in accordance with the Declaration of Helsinki and in compliance with national legislation.

## Author Contributions

KK, BH-D, EG, and MS-R designed the study. EG and GP collected the data. GP, MS-R, and NG performed statistical analysis. GP, MS-R, EG, GK, and TV wrote the manuscript. All authors read and approved the final manuscript.

### Conflict of Interest

The authors declare that the research was conducted in the absence of any commercial or financial relationships that could be construed as a potential conflict of interest.
