# Supplementary material for: Neural Correlates of Empathy in Boys With Early Onset Conduct Disorder
Source: Front Psychiatry. 2020 Mar 18;11:178. doi: 10.3389/fpsyt.2020.00178 (PMC7093593; doi:10.3389/fpsyt.2020.00178)
Supplement: Supplementary file 1 [file Data_Sheet_1.PDF]

Supplement 1

A Conjunction self > control – all participants

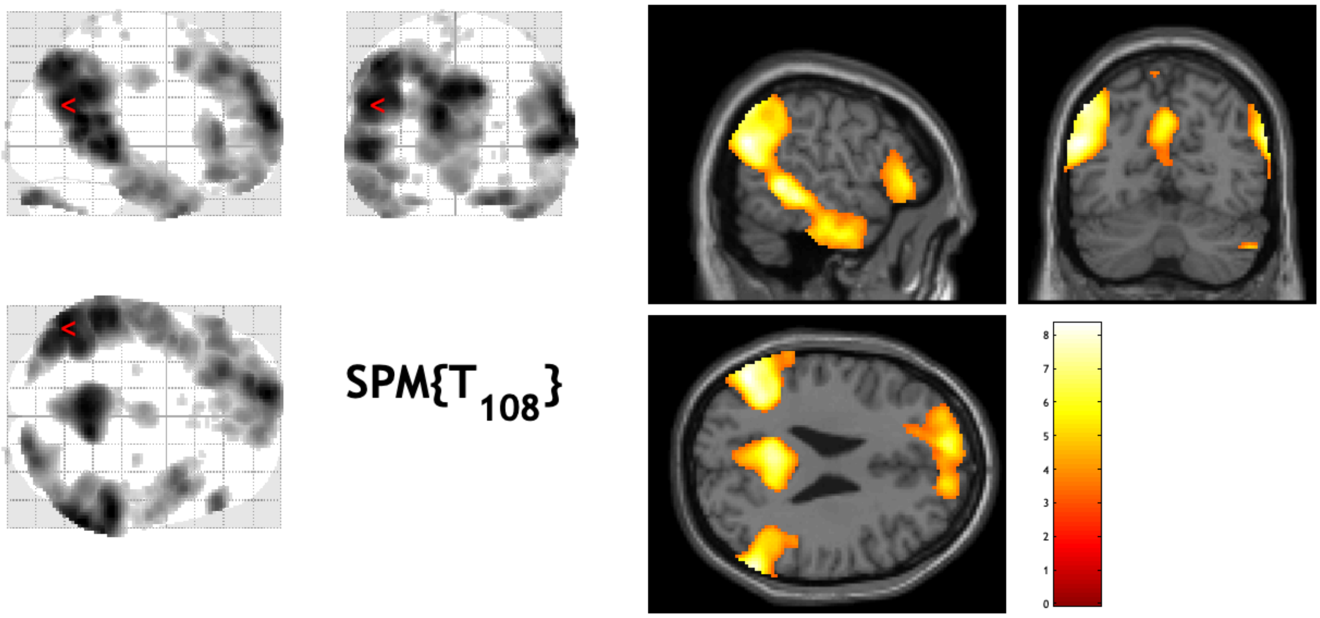

MNI-coordinates: -54, -66, 26; Glass brain (left) and SPM(T) overlaid on a single subject T1 template (right). For illustrative purpose, the height threshold was lowered to $p < 0.001$ uncorrected.

**Anatomical region**	**Cyto area**	**Side**	**MNI coordinates**			**Cluster size**	***T***	***P***
			**x**	**y**	**z**			
**Conjunction self > control**								
Middle Temporal gyrus	TE 3	R	**60**	**-36**	**0**	1575	**8,33**	<0.001
	PGa		66	-54	14		8,23	<0.001
			62	-62	26		7,76	<0.001
Lateral occipital cortex	PGp	L	**-54**	**-66**	**26**	3004	**8,26**	<0.001
Angular gyrus			-50	-66	48		8,10	<0.001
Middle temporal gyrus			-58	-44	-2		7,92	<0.001
Superior frontal gyrus		L	**-12**	**62**	**18**	1595	**8,20**	<0.001
			-18	50	38		7,23	<0.001
			-14	54	32		6,91	<0.001
Cingulum, posterior		L/ R	**-2**	**-52**	**32**	1387	**8,15**	<0.001
Precuneus			10	-50	34		8,02	<0.001
Cerebellum		R	**20**	**-88**	**-36**	172	**6,72**	<0.001
			28	-84	-34		6,67	<0.001
Inf. frontal gyrus, pars triang.	45	R	**54**	**30**	**0**	80	**6,42**	<0.001
Temporal pole		R	**46**	**8**	**-40**	104	**6,36**	**<0.001**

			40	4	-44		6,24	<0.001
Fusiform gyrus		L	**-58**	**-6**	**-28**	382	**6,00**	**<0.001**
Inferior temporal gyrus			-56	-20	-26		5,97	<0.001
			-48	-6	-32		5,73	0,003
Inferior frontal gyrus		L	**-44**	**30**	**-18**	93	**5,94**	**0,001**
Frontal orbital gyrus			-38	38	-20		5,41	0,009
Inf. frontal gyrus, pars triang.	45	L	**-54**	**30**	**0**	63	**5,81**	**0,002**
Temporal pole		L	**-46**	**2**	**-44**	30	**5,80**	**0,002**
			-40	12	-42		5,22	0,019
Middle frontal gyrus		L	**-42**	**10**	**50**	36	**5,57**	**0,005**
Superior frontal gyrus		R	**14**	**56**	**30**	51	**5,53**	**0,006**
Cerebellum		R	**52**	**-70**	**-38**	10	**5,46**	**0,008**
Gyrus rectus	FP2	L/ R	**-2**	**54**	**-22**	55	**5,34**	**0,012**
Medial front orbital	Fo1		2	48	-26		5,24	0,018

Note. Results for the conjunction analysis of self-condition > control-condition across groups. The Cyto area column indicates the assigned cytoarchitectonical area derived from the SPM ANATOMY toolbox v3.0 if available [Eickhoff et al., 2005]. All P-values are family wise error corrected for the whole brain (voxel-level). Clusters with less than 10 voxels are not reported.

B Conjunction other > control – all participants

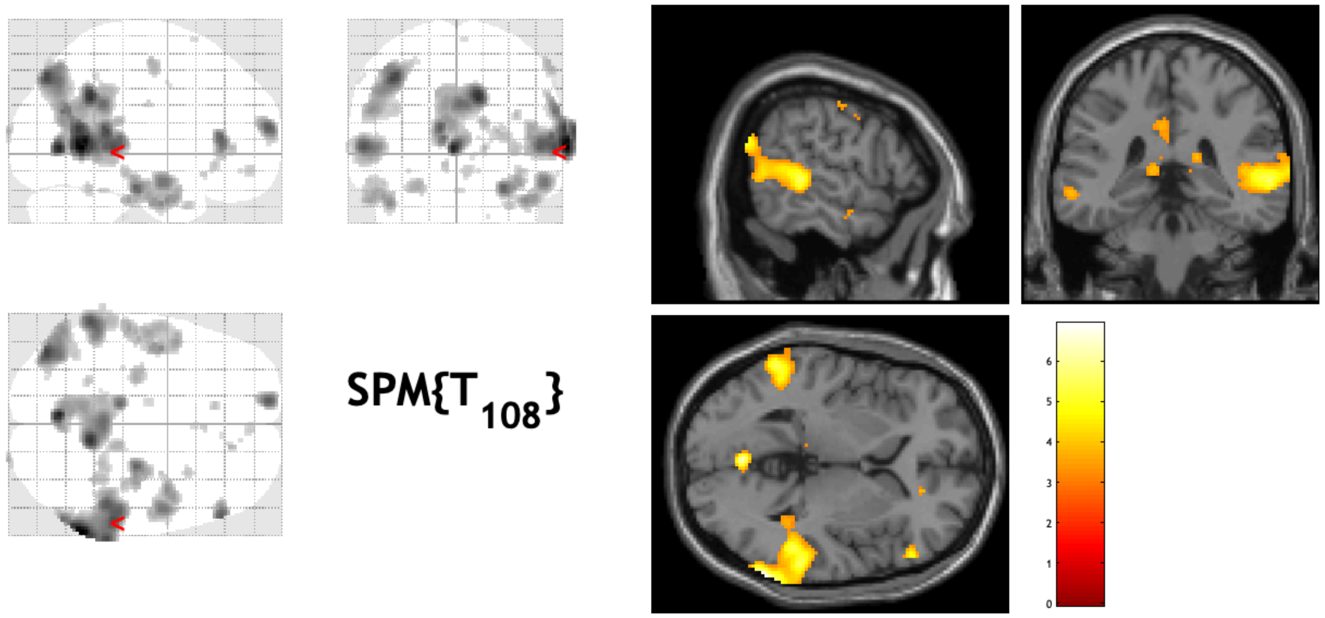

MNI-coordintates: 60, -36, 2; Glass brain (left) and SPM(T) overlaid on a single subject T1 template (right). For illustrative purpose, the height threshold was lowered to p < 0.001 uncorrected.

Anatomical region	Cyto area	Side	MNI coordinates			Cluster size	*T*	*P*
			x	y	z			
**Conjunction other > control**								
**Middle temporal gyrus**		**R**	**68**	**-56**	**2**	**120**	**6,92**	**<0.001**
Middle temporal gyrus/ BA 22			66	-58	14		5,78	0,002
Superior temporal gyrus			72	-40	12		5,55	0,005
**Lingual gyrus**	**V1**	**L**	**-4**	**-70**	**2**	**23**	**6,20**	**<0.001**
Posterior Cingulate		R	12	-50	32	39	5,93	0,001
Middle Occipital gyrus	PGp	L	-42	-78	44	31	5,63	0,004
**Middle temporal gyrus**		**R**	**60**	**-36**	**2**	**65**	**5,47**	**0,007**
			52	-40	4		5,32	0,013
Middle temporal gyrus		L	-60	-50	2	20	5,42	0,009
Medial frontal gyrus	p32	L	-12	62	14	7	5,31	0,014
Inf. frontal gyrus, pars triang.	45	R	58	32	8	4	5,21	0,020
Parahippocampus	Subiculum	R	28	-22	-22	2	5,15	0,025
Posterior Cingulate		L	0	-42	28	3	5,02	0,040

Note. Results for the conjunction analysis of other-condition > control-condition across groups. The Cyto area column indicates the assigned cytoarchitectonical area derived from the SPM ANATOMY toolbox v3.0 if available [Eickhoff et al., 2005]. All P-values are family wise error corrected for the whole brain (voxel-level).

C – single contrast TDC

1. Other - control

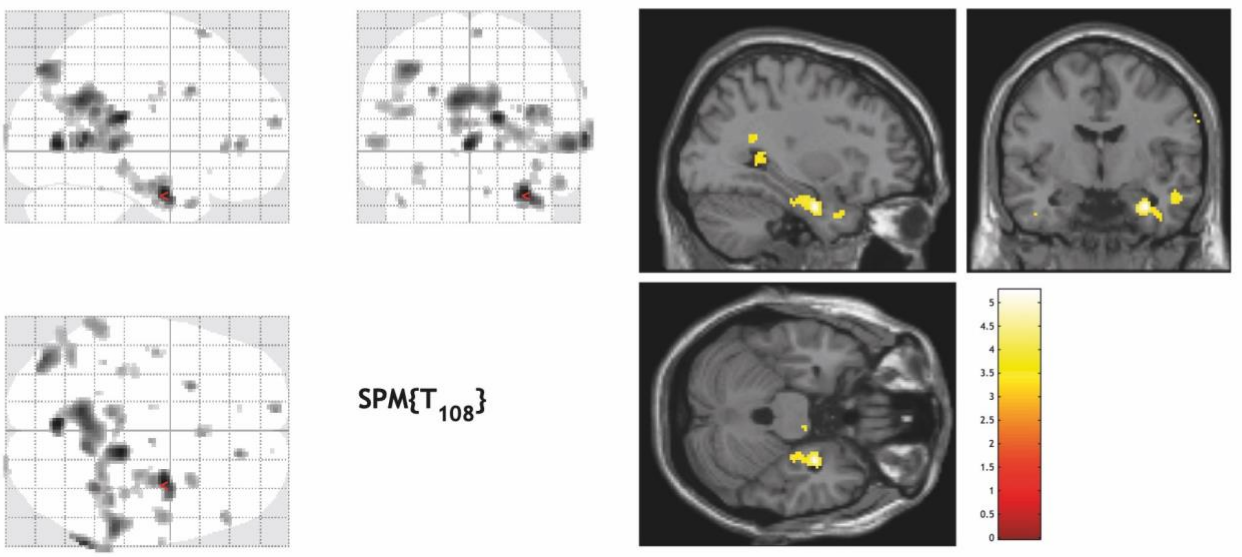

MNI-coordintates: 32, -6, -26; Glass brain (left) and SPM(T) overlaid on a single subject T1 template (right). For illustrative purpose, the height threshold was lowered to $p < 0.001$ uncorrected.

2. **Self - control**

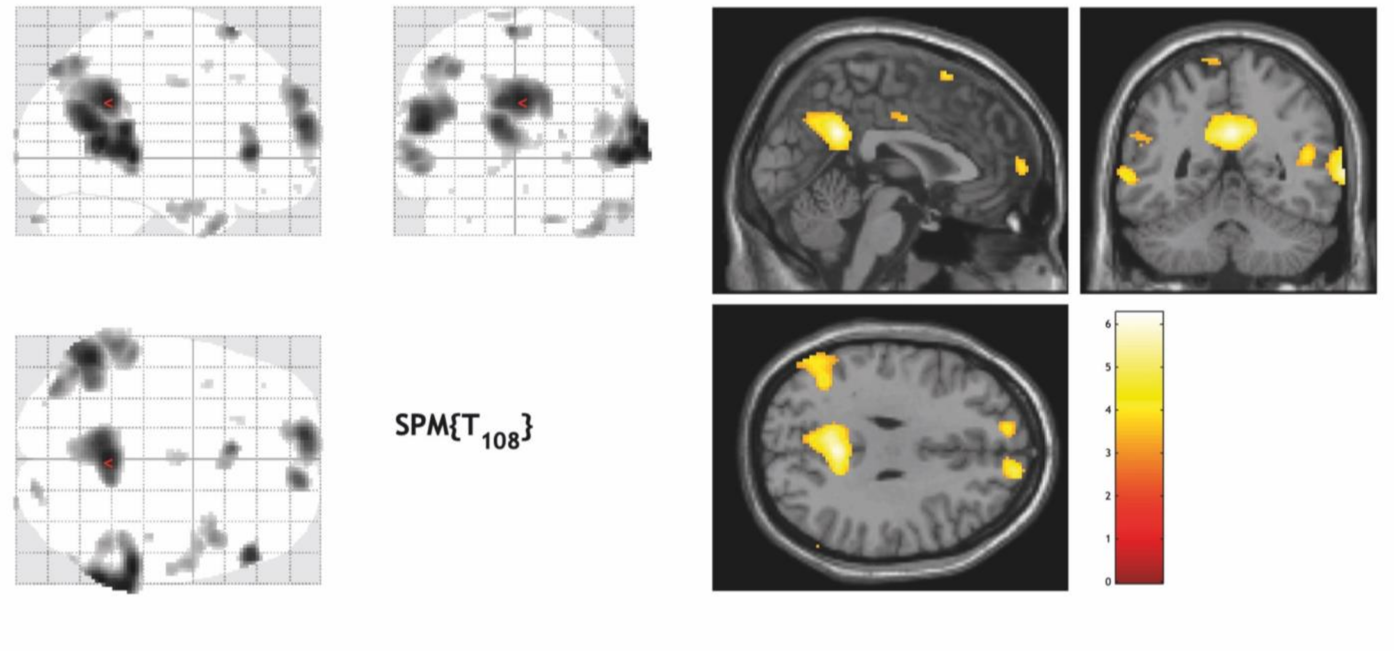

MNI-coordintates: 2, -50, 30; Glass brain (left) and SPM(T) overlaid on a single subject T1 template (right). For illustrative purpose, the height threshold was lowered to *p* < 0.001 uncorrected.

3. **Self – other**

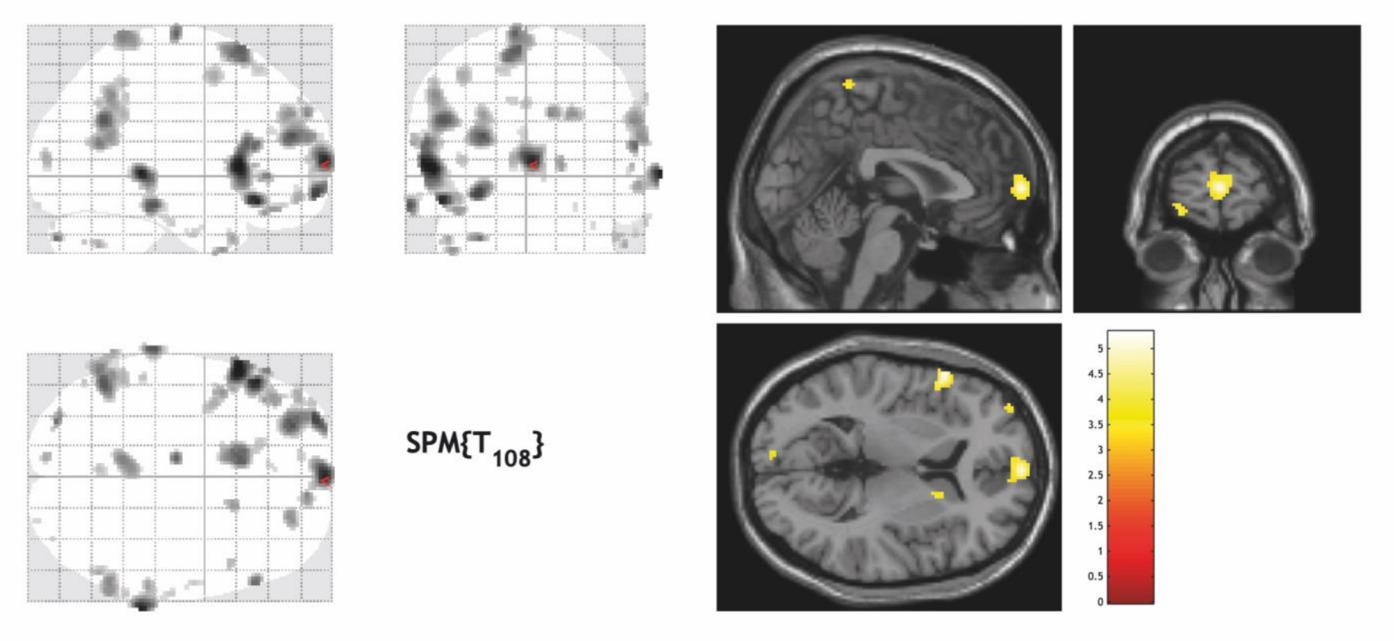

MNI-coordintates: 2, 66, 6; Glass brain (left) and SPM(T) overlaid on a single subject T1 template (right). For illustrative purpose, the height threshold was lowered to *p* < 0.001 uncorrected.

Anatomical region	Cyto area	Side	MNI coordinates			Cluster size	*T*	*P*
			x	y	z			
**other > control**								

Hippocampus		R	32	-6	-26	3	5.25	0.017
Lingual gyrus	Area hOc1 (V1)	L	-2	-70	2	2	5.15	0.025
**Self > control**								
Middle temp gyrus		R	68	-54	6	245	6.25	< .001
Angular gyrus		L	-54	-60	16	155	6.01	0.001
Post cingulate gyrus		R	2	-50	30	182	5.94	0.001
Inf. Front gyrus	Area 45	R	56	30	0	20	5.21	0.005
Frontal pole (med front)		L	-12	60	16	42	5.19	0.006
**Self > other**								
IFG, pars opercularis	Area 44	L	-58	18	4	15	5.00	0.014

Results for the analysis of other-condition > control-condition, self > control-condition and self > other condition within the typically developing group. The Cyto area column indicates the assigned cytoarchitectonical area derived from the SPM ANATOMY toolbox v3.0 if available [Eickhoff et al., 2005]. All P-values are family wise error corrected for the whole brain (voxel-level).

D – single contrast CD

1. Other - control

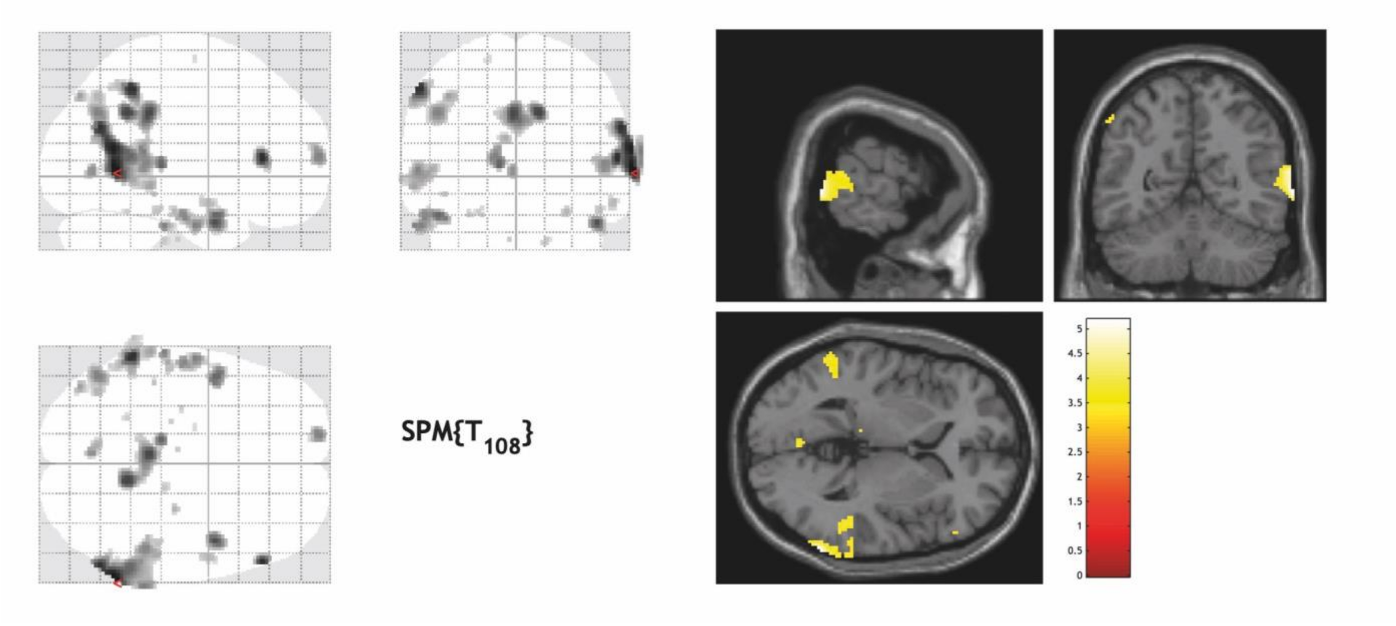

MNI-coordintates: 68, -56, 2; Glass brain (left) and SPM(T) overlaid on a single subject T1 template (right). For illustrative purpose, the height threshold was lowered to $p < 0.001$ uncorrected.

2. Self – control

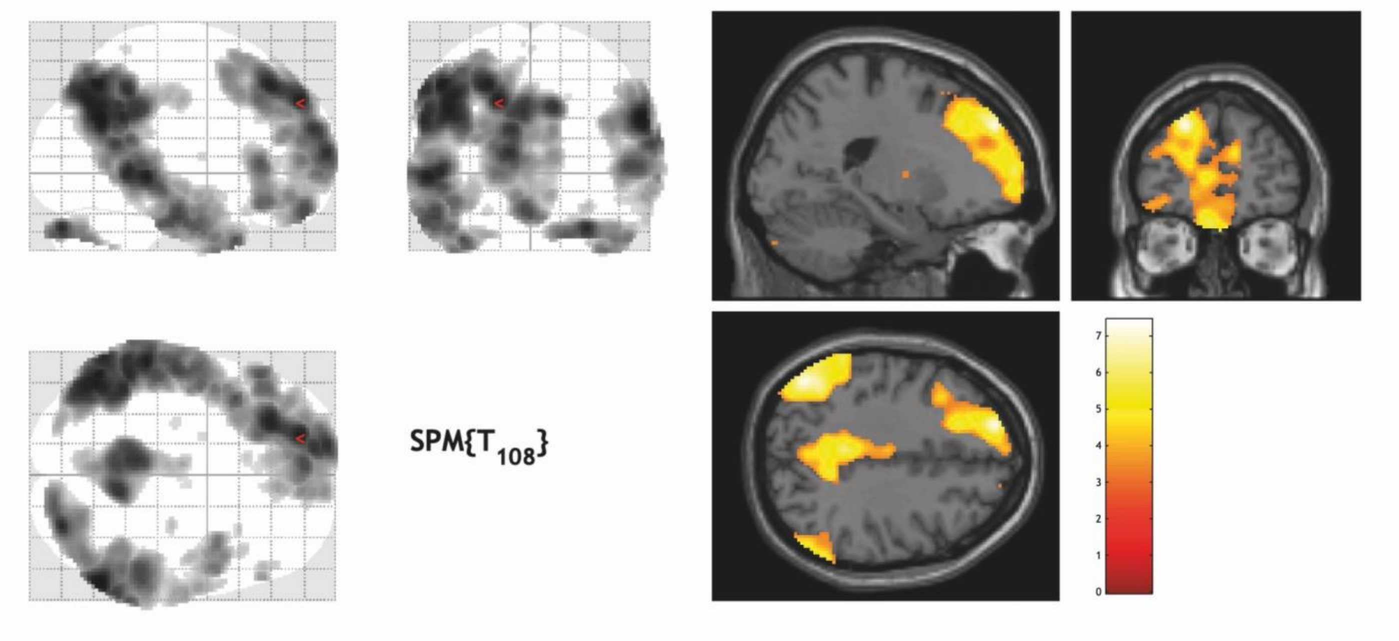

MNI-coordintates: -20, 50, 38; Glass brain (left) and SPM(T) overlaid on a single subject T1 template (right). For illustrative purpose, the height threshold was lowered to $p < 0.001$ uncorrected.

3. Self - other

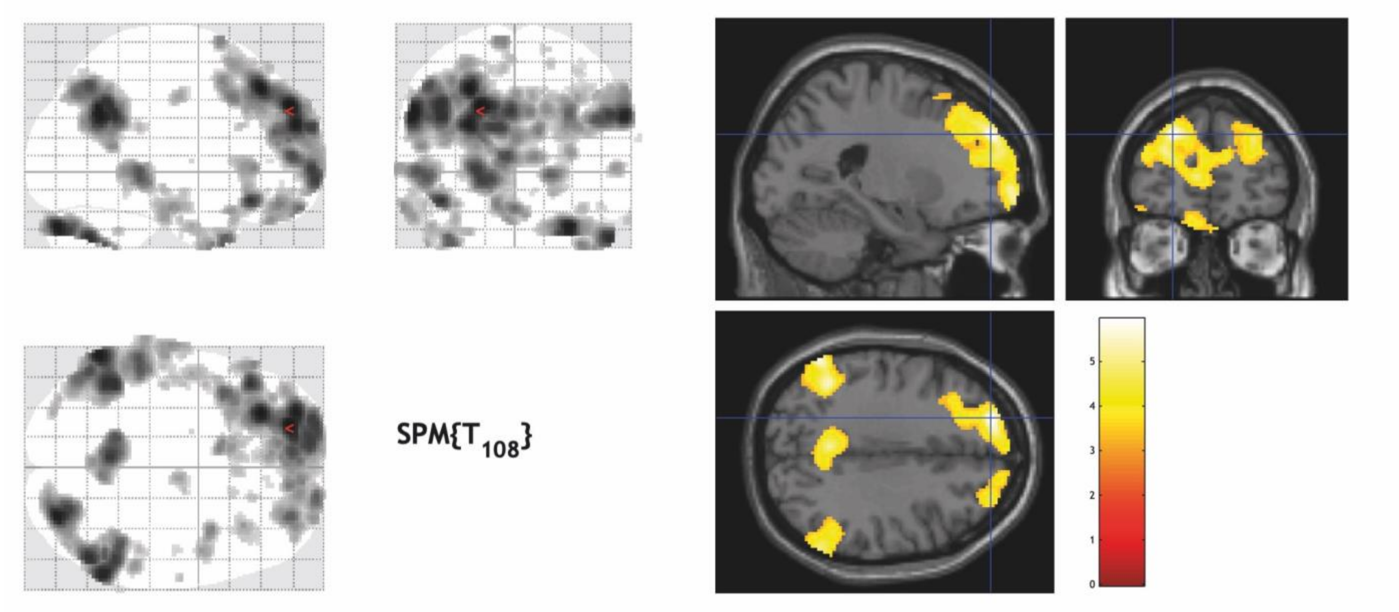

MNI-coordintates: -22, 50, 34; Glass brain (left) and SPM(T) overlaid on a single subject T1 template (right). For illustrative purpose, the height threshold was lowered to $p < 0.001$ uncorrected.

Anatomical region	Cyto area	Side	MNI coordinates			Cluster size	*T*	*P*
			x	y	z			
**other > control**								
Middle temporal gyrus		R	68	-56	2	4	4.88	0.023
**Self > control**								
Sup. Temp gyrus		R	62	-62	26	654	7.42	< 0.001
Angular gyrus	Area Pga (IPL)	L	-52	-64	46	1737	7.2	< 0.001
Middle temp gyrus, post devision		L	-60	-36	-4	610	7.09	< 0.001
Sup front gyrus		L	-20	50	38	1096	7.05	< 0.001
Cerebellum right, crus I		R	30	-84	-32	155	6.68	< 0.001
Precuneus/ post cingulum		R	12	-52	34	602	6.49	< 0.001
Inf temp gyrus, post devision		L	-56	-20	-26	327	6.22	< 0.001
Gyrus rectus	Area Fo1	L	-4	52	-24	84	5.99	< 0.001
Temporal pole		L	-40	14	-38	27	5.85	0.002
Inf frontal gyrus, pars triangularis	Area OP9/ Area 45	L	-52	28	4	38	5.79	0.001
Frontal orbital cortex		L	-46	28	-18	46	5.53	< 0.001
Right cerebellum crus 1		R	46	-72	-38	11	5.19	0.008
**Self > other**								
Middle/ sup frontal gyrus		L	-22	50	34	147	5.94	0.001
Middle frontal gyrus		L	-28	34	44	58	5.84	0.002
Angular Gyrus	Area PGa (IPL)	L	-60	-60	32	67	5.81	0.002
Cerebellum Crus I		R	28	-84	-32	57	5.76	0.002
Angular Gyrus	Area PGa (IPL)	R	62	-56	28	51	5.59	0.005
Angular Gyrus	Area hIP1 (IPL)	L	-44	-54	34	59	5.39	0.01
Cerebellum Crus I		R	52	-64	-40	16	5.36	0.012
Post Cingulate Gyrus / Precuneus		L	-4	-54	32	13	5.23	0.019
Sup frontal gyrus		L	-12	62	22	26	5.19	0.021
Middle frontal gyrus	Area Fp1	L	-24	64	0	28	5.18	0.023

Results for the analysis of other-condition > control-condition, self > control-condition and self > other condition within the conduct disorder group. The Cyto area column indicates the assigned cytoarchitectonical area derived from the SPM ANATOMY toolbox v3.0 if available [Eickhoff et al., 2005]. All P-values are family wise error corrected for the whole brain (voxel-level).

E – Behavioral analyses with consideration of high/ low intensity

In addition, correct identification of displayed emotions during empathizing was assessed based on correct responses during the other-task and analyzed by a by a 2x2x2 mixed-model ANOVA with the factors intensity, emotion, and group. We found a significant main effect of emotion, $F(1,27) = 12.06$, $p < 0.01$, due to a higher accuracy for sad ($M = 82.5.0\%$; $SD = 19.2$) compared to happy faces ($M = 71.0\%$; $SD = 16.5$; $p = 0.002$), and a main effect of intensity due to a higher accuracy in high ($M = 88.6\%$; $SD = 17.4$) vs. low intensity emotions ($M = 65.1\%$; $SD = 16.2$; $p < 0.001$). There was no main effect of group ($F(1,27) = 0.211$) and the interactions of emotion x group ($F(1,27) = 1.02$) and intensity x group x emotion were not significant ($F(1,27) = 0.37$). The interaction of intensity x emotion was significant ($F(1,27) = 42.6$, $p < 0.001$). Post-hoc testing across the whole sample indicated a more pronounced difference in accuracy for happy emotions with higher intensity ($M = 90.4\%$; $SD = 17.8$) vs. lower intensity ($M = 50.8\%$; $SD = 23.7$, $t = 8.4$, $p < 0.001$; Cohen's $d = 1.89$) compared to sad emotions (high intensity $M = 86.5\%$, $SD = 20.9$, low intensity $M = 78.5\%$, $SD = 20.5$, $t = 2.8$, $p < 0.05$, Cohen's $d = 0.38$). When restricting the analysis to low intensity, no group differences were detected (all $p > .05$).

Congruence of evoked emotions was assessed using congruent responses during the self-task (i.e. happy responses to happy faces and sad responses to sad faces) and analyzed by a 2x2x2 mixed-model ANOVA with the factors intensity, emotion, and group. We found a significant interaction of emotion x group ($F(1,27) = 7.1$, $p < 0.05$) and emotion x intensity ($F(1,27) = 13.3$, $p = 0.001$), and a main effect of intensity ($F(1,27) = 66.1$, $p < 0.001$); no main effect of group or emotion was observed (both $ps > 0.63$).

Post-hoc comparisons indicated a significantly higher congruency for sad ($M = 64.3\%$; $SD = 32.4$) compared to happy faces ($M = 51.4\%$; $SD = 27.4$; $p < 0.05$) in TDC but not in CD (p>.05). On a descriptive level, this pattern was reversed in CD (sad: $M = 44.2\%$; $SD = 38.2$; happy: $M = 60.8\%$; $SD = 29.2$). Further explorative post-hoc comparisons indicated a higher congruence for high intensity emotions ($M = 66.4\%$; $SD = 31.0$; $p < 0.05$) over low intensity emotions ($M = 44.1\%$; $SD = 26.7$; $p < 0.001$).

Restricting the analyses to low intensity, a main effect of group at a marginal level of significance was detected ($F(1,27) = 3.42$, $p = 0.07$). Exploratory post-hoc comparisons indicated a higher congruency of sad emotions over happy emotions in TDC ($M_{happy} = 33.1$, $SD = 28.9$, $M_{sad} = 55.8$, $SD = 34.4$, $t = 2.75$, $p = 0.02$), but not in CD ($M_{happy} = 45.5$, $SD = 28.9$, $M_{sad} = 41.5$, $SD = 39.1$; $t = -0.3$, $p > 0.7$).
